# Models of necessity

**DOI:** 10.3762/bjoc.16.137

**Published:** 2020-07-13

**Authors:** Timothy Clark, Martin G Hicks

**Affiliations:** 1 Computer-Chemistry-Center, Department of Chemistry and Pharmacy, Friedrich-Alexander-University Erlangen-Nürnberg, Nägelsbachstr. 25, 91052 Erlangen, Germany; 2 Beilstein-Institut, Trakehner Str. 7–9, 60487 Frankfurt am Main, Germany

**Keywords:** chemical bonding, chemical ontologies, chemical structure formats, chemical structure representation, chemical structure models, language of chemistry, quantum chemistry

## Abstract

The way chemists represent chemical structures as two-dimensional sketches made up of atoms and bonds, simplifying the complex three-dimensional molecules comprising nuclei and electrons of the quantum mechanical description, is the everyday language of chemistry. This language uses models, particularly of bonding, that are not contained in the quantum mechanical description of chemical systems, but has been used to derive machine-readable formats for storing and manipulating chemical structures in digital computers. This language is fuzzy and varies from chemist to chemist but has been astonishingly successful and perhaps contributes with its fuzziness to the success of chemistry. It is this creative imagination of chemical structures that has been fundamental to the cognition of chemistry and has allowed thought experiments to take place. Within the everyday language, the model nature of these concepts is not always clear to practicing chemists, so that controversial discussions about the merits of alternative models often arise. However, the extensive use of artificial intelligence (AI) and machine learning (ML) in chemistry, with the aim of being able to make reliable predictions, will require that these models be extended to cover all relevant properties and characteristics of chemical systems. This, in turn, imposes conditions such as completeness, compactness, computational efficiency and non-redundancy on the extensions to the almost universal Lewis and VSEPR bonding models. Thus, AI and ML are likely to be important in rationalizing, extending and standardizing chemical bonding models. This will not affect the everyday language of chemistry but may help to understand the unique basis of chemical language.

## Introduction

This article originally arose out of our discussions for the 2020 Beilstein Bozen Meeting “*Models of Convenience*” [1], which had to be postponed due to Covid-19 pandemic. It essentially contains the content of the first two presentations, which were intended to set the scene for the remainder of the workshop. We have decided to publish it in the present form to provide a starting point for the postponed workshop, whatever form it may take.

Over the centuries, humanity has tried to explain natural and physical phenomena through theories and models. These fitted ever more facts as understanding grew and methods of measurement became more sophisticated. It is only just over 200 years ago that the caloric theory of heat, which regarded heat as a fluid, was challenged and began to be replaced by a theory associating heat with motion [2]. The discovery of Brownian motion by Robert Brown in 1827 [3], followed far later by Einstein’s paper on the molecular kinetic theory of heat in 1905 [4], showed that temperature was directly linked to molecular movement. In contrast to the common picture of a sudden scientific revolution when a new theory appears, changes like this can often take a century. Prominent scientists often reject a new theory: Despite the successes of quantum theory, Einstein was convinced that it was nevertheless not a complete description of reality [5]. The “truth” is seldom a criterion for the acceptance of a model: Our current models of atoms and molecules are very useful and necessary for communicating but possess strong model character [6]. They break down, for example at very high pressures, where the standard bond orders of atoms change dramatically [7]. Physical properties at the nanoscale are often not understandable in terms of our usual descriptions, just as Newtonian mechanics are fine for the billiard table but at the atomic and astronomical scales insufficient. Models are needed for communication and understanding, but models are incomplete, and understanding is a subjective attribute.

**Figure 1:**
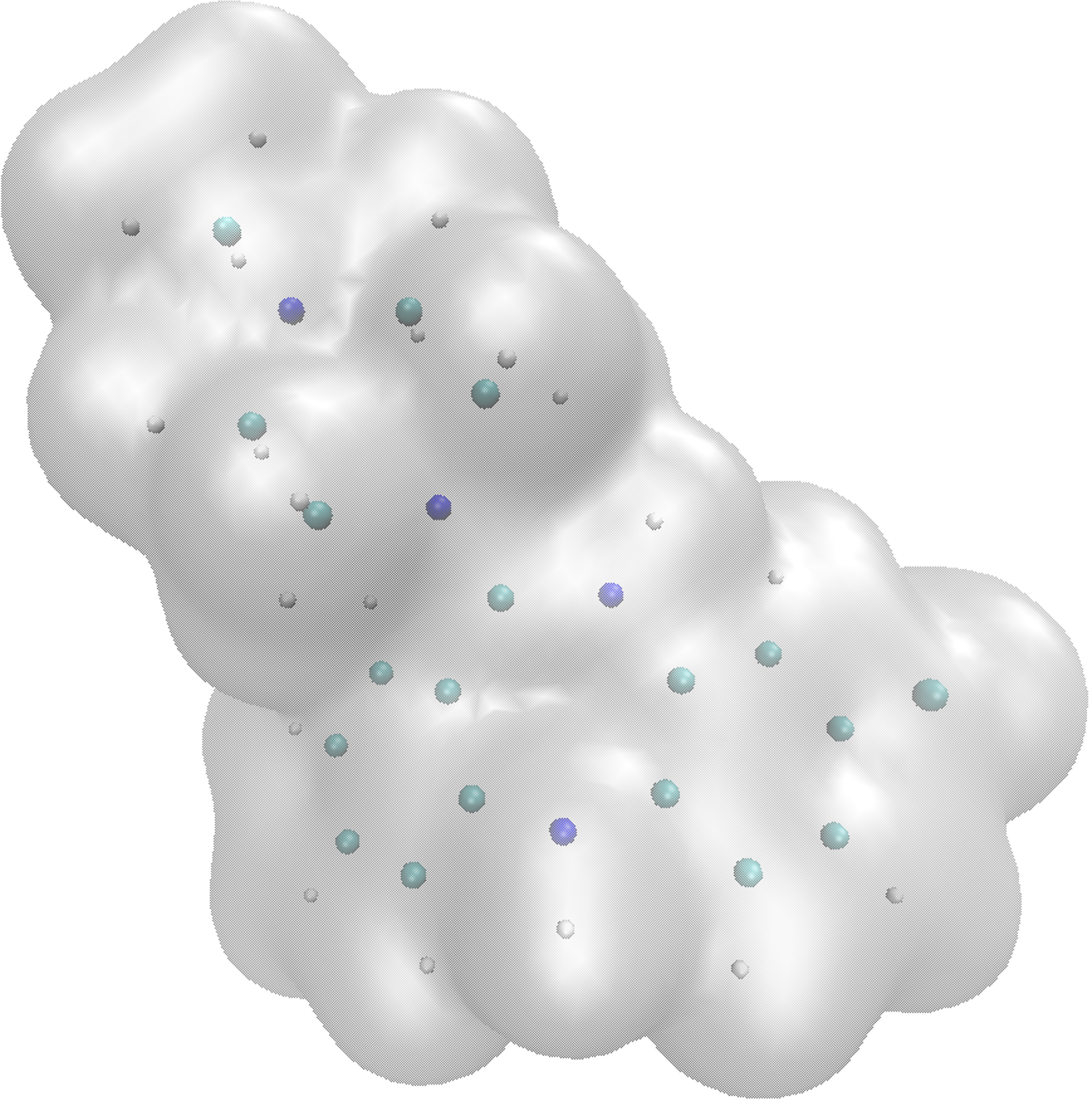
Representation of clozapine to emphasize the quantum mechanical characteristics of the molecule. The nuclei are represented as spheres (whose size is vastly exaggerated) color coded according to their charge. The gray surface is approximately the 0.01 a.u. isodensity surface, which corresponds approximately to the van der Waals surface of the molecule.

Chemistry is unique among the natural sciences in that its everyday systematization and interpretation depend almost entirely on simplified models of molecules. This is because the “truth” is unwieldy and, for a science that routinely deals with many millions of molecules, completely intractable. We have placed the word “truth” in quotation marks because of its complex connotations in this discussion [8]. What we mean is that neither wave functions nor electron densities can serve as framework models for chemistry in general; they are too complex. Figure 1 shows an “artist’s impression” of a molecule (clozapine) that illustrates the problem. In a puritanical quantum mechanical view, molecules consist of positively charged, and within the Born–Oppenheimer approximation [9] static, nuclei within a cloud of indistinguishable electrons, which are often represented as an electron-isodensity surface [10], as in Figure 1. Importantly, there are no atoms and no bonds within the molecule, only nuclei and indistinguishable electrons.

Although it is conceptually accurate, Figure 1 shows a picture of molecules that is completely impractical for a systematic science. Chemistry needs to be able to define molecular structures uniquely within a common language and to use this language to develop a classification scheme that can deal with many millions of different molecules (compounds) and, even worse, reactions. This need is more fundamental than looking for a representation of molecules suitable for depiction in databases, cheminformatics, machine learning (ML) or artificial intelligence (AI): It is essential for chemists to be able to communicate with each other about molecules. The language of chemistry varies slightly between the organic and inorganic communities. However, it is always based on bonds, functional groups, atomic centers and ligands, none of which appear in Figure 1.

It is important here to distinguish between what are essentially three different languages, or ontologies, of chemistry. The first and best known is the mixture of different bonding and orbital concepts that serve for daily communication between experimental chemists and often as a conceptual basis for their research. This language is fuzzy and varies from chemist to chemist but has been astonishingly successful and perhaps contributes with its fuzziness to the success of chemistry. It is this creative imagination of chemical structures that has been fundamental to the cognition of chemistry and has allowed thought experiments to take place. We should never forget that the golden age of German organic chemistry at the beginning of the 20th century happened almost a century before quantum mechanical calculations became a standard tool and even a decade before Lewis’ bonding model. At the other extreme is the purist quantum mechanical view exemplified by Figure 1. This “language” is able to reproduce structures, energies, reactivities, optical properties etc. of chemical compounds essentially perfectly if we do it well enough. However, it is utterly useless as an everyday language of the subject unless we attempt to translate it into one of our bonding models; a language that has no inherent definition of atoms or bonds is not suitable for talking about molecules or reactions. Weisberg [11] describes this situation as using a so-called folk ontology (the everyday language of chemistry) as a fiction when discussing a very complex mathematical model (quantum mechanics). The third ontology, and the one that interests us most here, is how we describe molecules to computers for building databases, developing property prediction algorithms and now for ML and AI applications. In this case, external conditions apply that lead to a different ontology than the two others. We discuss some features of these languages in the following and relate them to existing concepts.

**Figure 2:**
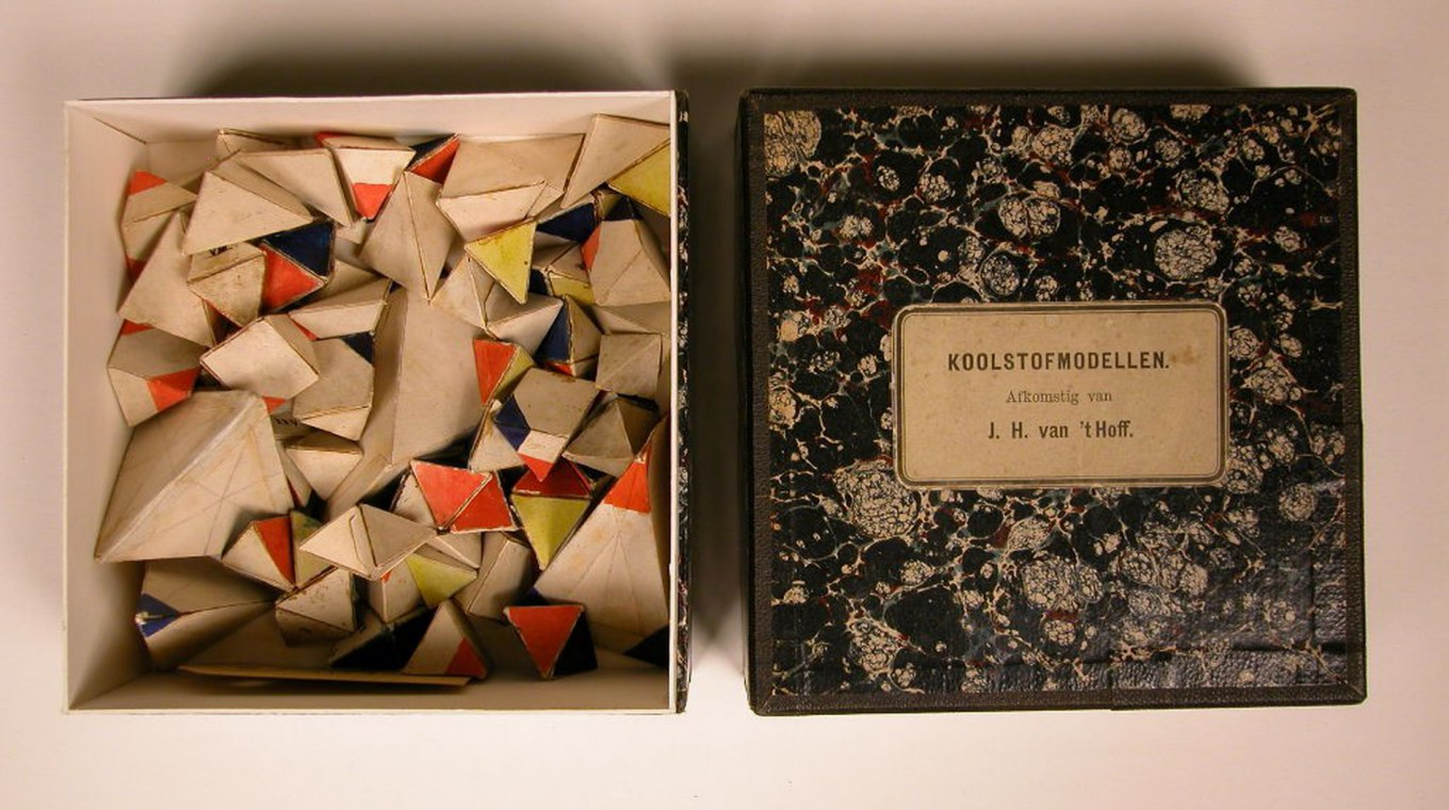
Jacobus van’t Hoff’s molecular models. The photography was reproduced from: https://rijksmuseumboerhaave.nl/steun-het-museum/particulieren/schenken/geadopteerde-objecten/ (access date 19.06.2020) with permission from the Rijksmuseum Boerhaave. © the National Museum Boerhaave, Leiden. For any reproduction for this photography the National Museum Boerhaave, Leiden has to be asked for permission.

The title “Models of necessity” indicates that such concepts, which are conceptual models, make the systematic study of chemistry possible at all. One early and historically important model is that of van’t Hoff, which really was expressed in the physical models shown in Figure 2 [12]. As outlined below, a consistent workable framework model, or more accurately series of models, has developed in chemistry.

The proliferation of AI and ML in chemistry will make new and far more extensive use of our models but will also impose new constraints and requirements on them that eventually will benefit all of chemistry by imposing a stricter intellectual discipline on the models used. We note here that the expressions “models”, “ontology” and “language of chemistry” are in this context equivalent.

## Discussion

### The underlying model; the Lewis picture

**Figure 3:**
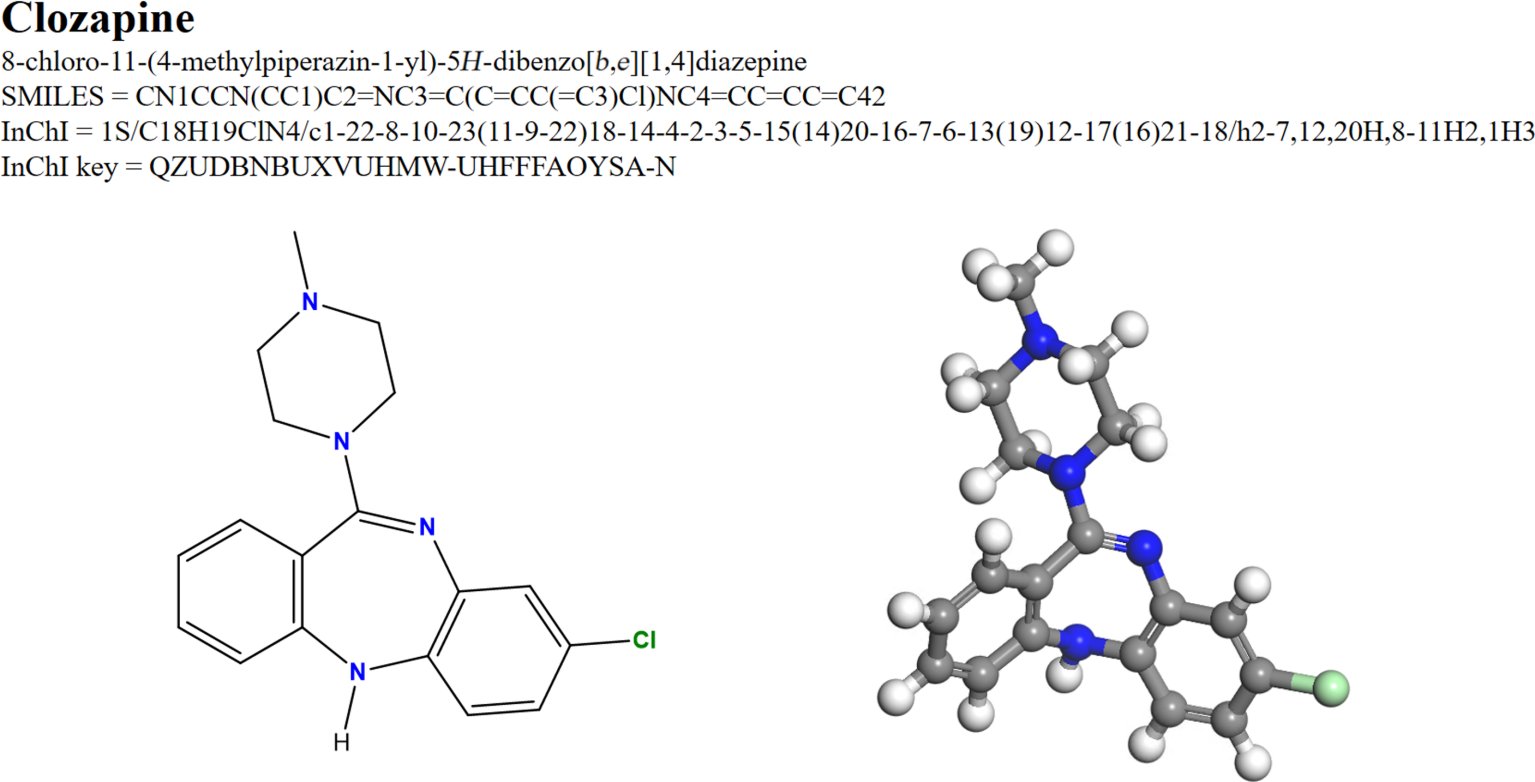
IUPAC name, canonical SMILES, InChI and InChI key, Lewis structure (2D line diagram) and 3D ball-and-sticks model for clozapine, the molecule shown in Figure 1. Data taken from https://pubchem.ncbi.nlm.nih.gov/compound/Clozapine#section=IUPAC-Name.

Although van’t Hoff’s models provided a major step forward in the theory of molecular structures, they are poorly suited for written communication. This function is fulfilled almost ideally by Gilbert Lewis’ structural formulae [13]. Lewis drew on a long history of depicting molecules in terms of their constituent atoms [14] and added a rationalization in terms of stable electron octets that survives in an extended form to this day and bears his name. Not only do these formulae provide the basis for fundamental concepts such as functional groups but also for just about every cheminformatics representation of molecular structures. Figure 3 shows the Lewis formula of clozapine (the molecule shown in Figure 1) together with its IUPAC systematic name [15], SMILES [16] and InChI [17] keys.

Chemists immediately recognize the aromatic and heterocyclic rings, the piperazine ring and the chloro-substituent in the 2D structure shown in Figure 3. Although the atomic coloring is not universal, most chemists would also recognize that the nitrogen atoms are blue and the chlorine green in the ball-and-sticks model, so that the atomic labels are not necessary. Even the alternating double and single bonds in the benzene rings are recognized to indicate aromaticity. Chemists immediately know where the molecule is most likely to be protonated or deprotonated and where the aromatic rings should be substituted, all from the Lewis structure. Many cheminformatics practitioners would also be able to write the structure from the SMILES string and medicinal chemists to recognize it as a benzodiazepine and assign it to a likely therapeutic area. The point is that these depictions are very different to the puritanical “truth” shown in Figure 1 but that they provide the basis to discuss and classify the molecule and to estimate its chemical, physical and even medicinal properties. As an aside, note that the 3D ball-and-sticks model only represents one of several possible conformations of clozapine but that all these conformations are inherent in the 2D-line structure.

The dichotomy, really that between fundamental quantum chemistry and the common bonding models, which is made evident in Figure 1 and Figure 3, is the subject of this article. It means that just about all practical chemistry and most data-processing applications in chemistry depend entirely on bonding models that are not inherent in the, as far as we know correct, quantum mechanical description of the model.

In reality, even the quantum description is usually an approximation because it is often applied to single molecules and not to the reality of phases containing ensembles of the molecule in question, solvents, counterions, reagents etc. Simplifications and approximations within the quantum mechanical technique add another layer of imperfection. Biological systems are even more complex, with an extraordinarily large concentration of molecules in, for instance, cells. In such systems, the necessity to sample all possible geometrical configurations of the system becomes the second major hurdle to accurate simulations. Even today, high-level combinations of an accurate Hamiltonian to describe the structure and energy and adequate sampling of the dynamics of the system are extremely rare.

### Atoms, bonds and molecules

Although there are no atoms or bonds in molecules, there have been many attempts to construct Lewis-like bonding pictures from quantum mechanical wave functions or electron densities. The first were so-called population analyses such as those of Coulson [18] or Mulliken [19] that assigned net atomic charges or bond orders. Ideally, single bonds in the Lewis picture should have a bond order of one, double bonds of two, and so on. The concept of net atomic charges stems directly from Lewis’ polar covalent bond concept but has no unique definition for the fundamental reason that individual atoms are not uniquely defined within molecules.

Although the positions of the atomic nuclei are clearly defined, the cloud of indistinguishable electrons prevents assignment of electrons, or fractions of them, to individual nuclei to make up atoms. Nonetheless, there are two principal methods to assign electron density to a given nucleus, both to a certain extent arbitrary and both subject to many variations.

The largest group of methods is based on the Linear Combination of Atomic Orbitals (LCAO) approximation [20]. LCAO is strictly speaking an approximation; molecular orbitals are built as weighted sums of constituent atomic orbitals, usually centered at the positions of the nuclei. This very convenient approximation for computational quantum chemistry has long attained the status of a bonding model to such an extent that many chemists outside the theoretical community are unaware that it is an approximation. Molecular-orbital bonding models based on the LCAO approximation discuss chemical bonds in terms of contributions from atomic orbitals. The inherent assumption in these techniques is that bonding contributions by orbitals centered on a given nucleus can be assigned to the corresponding atom. This assumption can be defended for basis sets (the atomic orbitals) that are small and localized but high-quality calculations use very space-extensive atomic orbitals (AOs, basis functions) that extend into the neighborhood of adjacent atoms (however that is defined). This problem manifests itself concretely as basis-set superposition error (BSSE) [21] in ab initio calculations but rears its ugly head in every AO-based attempt to assign electron density to individual atoms. It casts doubt on techniques that rely on partitioning electron density on the basis of AOs, although techniques such as the Natural Population Analysis (NPA) [22] or Absolutely Localized Molecular Orbital (ALMO) [23] analyses have been developed to give more constant results as the extent of the basis set increases. The basic problem, however, remains that where a basis function is centered in space has little to do with how to assign electron density to notional atoms.

The second, smaller group of analysis techniques relies on partitioning space around the nuclei into different volumes that can be assigned to individual atoms. The most prominent of these is Bader’s Quantum Theory of Atoms in Molecules (QTAIM) [24] which provides a consistent definition of the partitioning between atoms based on the topology of the electron density. QTAIM is well defined (but still arbitrary) but has been criticized because it sometimes gives counterintuitive atomic charges, for instance. Extensions to the partitioning of the space around atoms, such as bond-critical paths, are not accurate descriptions of molecular structures [25]. The current situation is that each technique has its followers, often on the basis that it gives results with which the users feel comfortable. It is hard to believe that such criteria are objective and unique.

### Molecular orbitals

Two bonding models exist side by side in chemistry, most obviously in organic chemistry. Alongside the Lewis picture and its mechanistic “curly arrow” treatment [26] it is common to rationalize organic structures and reactivities within the molecular-orbital picture, as pioneered by Walsh [27] and Fukui [28]. Elementary organic chemistry is taught almost exclusively within the “curly arrow” model until it is necessary to switch to the molecular-orbital picture to treat, for instance, the Woodward–Hoffmann rules [29]. Similarly, an extended version of the Lewis model exists alongside the molecular-orbital picture in inorganic chemistry. Is this not a solution to the problems described above?

Unfortunately not: Molecular orbitals (MOs) were introduced by Mulliken [30] as an approximation in order to be able to solve the Schrödinger equation approximately for atomic or molecular systems. The approximation is that, rather than trying to solve a completely intractable *N*-electron problem, we solve *N* easier one-electron problems. The resulting one-electron wave functions were named molecular orbitals by Mulliken. Thus, they are not real, but another approximation.

How, then, do we account for the success of qualitative MO theory if it is based on fictitious one-electron wave functions? The answer is that the commonly used Hartree–Fock MOs strongly resemble Dyson orbitals [31] which are real and measurable [32]. This observation provides a link between MO theory and real observables, so that qualitative MO theory is a widely used and successful approach to understanding chemical structures and reactivity. However, it is too unwieldy for everyday communication.

### Qualitative bonding concepts

The basic conundrum remains. If we cannot define atoms within molecules, we cannot define atomic charges or bonds between atoms or interatomic concepts such as charge transfer. It is important to emphasize here that this fundamental problem cannot be resolved because of the nature of the quantum mechanical wave function or electron density. All models that aim to derive Lewis-like concepts from quantum mechanical calculations are therefore arbitrary, however reasonable they may seem, and can only be judged on how well the results fit with the user’s own preferences and prejudices.

**Figure 4:**
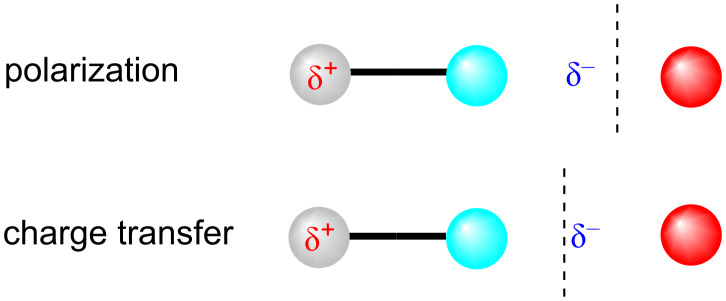
Conceptual distinction between polarization and charge transfer. The distinction depends entirely on the definition of the atoms, and even the bond. In both cases, the result is the same increase in dipole moment. The vertical dashed lines indicate notional borders between atoms. The two phenomena are identical if neither atoms nor bonds can be defined.

Take, for instance, the concepts of polarization and charge transfer. Of these two closely related concepts, polarization is real and observable (e.g., through a change in dipole moment), whereas charge transfer is a qualitative concept that can neither be defined nor observed uniquely. This is illustrated in Figure 4. Polarization (above) involves a shift of electrons from the gray atom to the cyan one; charge transfer from the gray/cyan molecule to the red atom. The charge shift in the two examples is identical. Simply moving the black dashed borderline changes the definition from polarization to charge transfer. In a simple, Lewis-like picture, the distinction is clear but quantum mechanically the two processes are the same. Figure 4 shows that the difference between the two lies in the definitions of the atoms (and hence net atomic charges) and of the bond. Physically, both result in the same change in dipole moment. Objectively, they are not distinguishable, as has been pointed out many times [33].

### Quantum mechanics to Lewis or the reverse?

The preceding section could easily be expanded into a complete book but extracting Lewis structures and assigning bonding contributions from quantum mechanical calculations is actually a minority pursuit most often used to rationalize experimental results. Should the conversion between quantum mechanical calculations and Lewis structures be necessary, it is usually in the reverse direction: Lewis structures are drawn or defined by a line notation such as SMILES, converted by one of several algorithms into a realistic 3D molecular structure and the resulting structure used as input for a quantum mechanical calculation. What quantum mechanics does far better than rationalize results in terms of the Lewis picture is to provide hard, physically observable properties of molecules [34] such as structures, energies (and energy differences), molecular electrostatic potential in space [35], polarizability [36] electronic spectra [37] or reactivity. Some of these properties may then be used in cheminformatics applications to derive structure–property relationships for partition properties or solubility or structure–activity relationships for biological activity. Note, however, that the non-physically-observable model properties such as net atomic charges [18–19,38–39] are used far more often as descriptors in this context, although the adequacy of these descriptors for reaction prediction has been questioned [40].

### The role of models in describing molecules and reactions for AI and ML

Only thirty years’ ago, before the advent of everyday computer graphics programs and tools that allow chemical structures to be displayed and manipulated in 2D and 3D, most chemists used small physical model building sets, the successors of van’t Hoff’s models, that allowed them to create 3D structures of molecules of interest [41]. These were very useful for solving issues of conformation, reactivity and structure, for example in the determination of the structure of DNA [42]. In inorganic chemistry, the standard Lewis eight-electron picture is complemented by the VSEPR (Valence Shell Electron Pair Repulsion) or Gillespie–Nyholm model [43], which gives rise to a series of standard geometrical shapes that describe common coordination patterns (linear, trigonal planar, tetrahedral, trigonal bipyramid and octahedral) around central (usually metal) atoms. Neither the Lewis nor the VSEPR model is universally successful but they provide a conceptual basis for understanding and communicating ideas about chemical structures and reactions. This early extension of the Lewis model and the introduction of two-center three-electron bonding [44] serve as prototypes for what will become necessary improvements to handle in particular non-covalent interactions but in general any structures or interactions not treated by the existing model.

Chemistry and chemists need to describe more than static molecules: A large part of the science is devoted to reactions, in which molecules combine or dissociate and result in new, different molecules. On the one hand, combining two or more poorly described entities (molecules) exacerbates the situation, multiplying the inadequacies. On the other hand, since chemists describe reactions in terms of bonds made or broken, the Lewis model lends itself well to describing both molecules and reactions. Furthermore, as with single structures, the storage and searching of reactions in chemical databases, based on connectivity of atoms has become a very useful tool for the synthetic chemist. We have mentioned one limit above; that the Woodward–Hoffmann orbital-based rules are necessary to describe electrocyclic reactions [29].

Reaction databases that only store reactions are relatively straightforward constructions, if we look further, for example to systems for predicting reactions or suggesting synthetic routes [45], whether using manually coded transformations or developments using automated machine learning and AI techniques, limitations of the Lewis model become apparent [40]. This is because non-equilibrium structures such as transition states that cannot be described by the conventional Lewis model are passed through during reactions. Quantum chemical methods, often in combination with molecular dynamics, can predict the course of a specific reaction accurately but at a high cost in human and computer time for all but the simplest reactions. Additionally, reactions do not usually occur for isolated molecules but in solution, on a surface or within porous solids, at high temperatures or pressures. These conditions can seldom be considered completely in computational studies. Furthermore, reactions often follow competing paths, where the dominance of one over another often depends on the specific reaction conditions and only minor variations can give rise to a different outcome. Biological systems have a network of pathways and it is often very difficult to determine which path predominates.

Optimizing reaction conditions is often down to trial and error. In this regard it is worth noting that a major deficit in information lies in the scope of a particular reaction and of negative results; i.e., reactions that do not work. Until there are sufficient standardized data available regarding variations in the parameters for a reaction (e.g., temperature, pressure, pH, solvents, reagents, catalysis etc.) it will be difficult to use AI/ML methods successfully and routinely. What is needed is true Big Data, where real-time data is captured from laboratories carrying out experiments under defined conditions. Currently, several research groups are working on the area of automated syntheses so that not only the reactions and workup are automated, but also the conditions of the reaction closely monitored, controlled, varied and automatically recorded. For examples, see [46–49]. A convergence of technologies, such as robotics, telemetrics, analytics, in addition to using ML/AI techniques, could allow an infrastructure to develop that enables data-driven discovery [50] and transforms chemistry from an empirical to a predictive science [51].

Computer systems for predicting reactions or suggesting synthetic routes have been developed since the 1960s. They usually differ in one of two ways: They either involve considerable work in manual annotation of the transformations, as with the seminal work of Corey and Wipke with LHASA (Logic and Heuristics Applied to Synthetic Analysis) [52], or implement an automatic ML approach that encodes the reactions based on a training set, as pioneered by Gelernter [53–54]. Latterly, hard- and software improvements have made advances possible (see, for example [55–57]), as has an unprecedented effort in the manual coding of reactions for Chematica [58]. This work has shown that for many cases of reactions, the information implicit in the molecular graph is insufficient to give necessary discrimination [40]. Thus, electron densities or wave functions are needed. While such information can be calculated routinely using quantum mechanics, the effort and time involved makes this impractical for large numbers of molecules, and impossible for the hundreds of millions of known molecules. Program developers [56] have gone back to methods developed in the 70s and 80s such as the Gasteiger–Marsili [38–39] method for calculating partial charges. Such methods, although seemingly outdated, are uniquely fast because of the limits in computer power when they were developed. Chematica [58] uses among other parameters the Hammett substituent constant [59] developed in the 1930s. These uses of group contribution methods, or of very approximate calculations in general, suggests that, in many areas of cheminformatics, the size of the data generated has outgrown the methodology to process it effectively. This conclusion, however, may be premature. Fast and approximate quantum mechanical techniques provide alternatives, as was shown as long ago as 1998 [60], admittedly for “only” 53,000 molecules.

A problem is that chemistry is not a traditional data science; a major difference between chemistry and other scientific disciplines is that chemists continuously create new research entities; molecules. Thus while each new molecule is a piece of new knowledge that increases the known chemical space, the interaction of new molecules with those previously known is essentially unknown and will in general not be investigated: Chemists are in fact increasing the relative lack of knowledge. Largely, chasing after the new is prioritized over fully investigating the known. This is exemplified by the fact that for many researchers, the most valuable search in a large chemical structure database is often one that retrieves (correctly) zero hits.

Cheminformatics is an old discipline that preceded the current interest in ML and AI by several decades, as shown by the subject of the 2000 Beilstein Bozen Symposium [61]. A recent discussion of AI and ML in chemistry and drug design [62] traces the beginning back to the classical 1964 papers of Hansch and Fujita [63] and Free and Wilson [64]. All that has really changed recently is the amount of data that is available and the upsurge of deep-learning algorithms, which date back to the late 1960’s [65] but were preceded in chemistry by far simpler back-propagation neural nets [66] and made their first impact around 2015 [67]. At the second Beilstein Bozen Symposium in 1990, Prof. Gerald Maggiora described a then novel use of a back-propagation neural net to predict the products of organic reactions [68] and Prof. Herbert Gelernter the use of inductive and deductive machine learning to build a knowledge base for synthetic organic chemistry [54]. It is therefore not surprising that the problem of describing molecules to computers was essentially solved more than thirty years ago. The first simple line notations eventually gave way to SMILES [16], which is still very widely used, and later to the less instinctive but more powerful InChI [17]. These line notations and connection tables [69] are based entirely on a Lewis bonding picture. Indeed, there is no need to use the chemical structure to generate descriptors for modeling, structure–activity and structure–property relationships, the forerunners of today’s ML approaches. SMILES strings themselves and fragments thereof serve equally well as descriptors [70]. Thus, with very few exceptions based on physical observables such as the molecular electrostatic potential [35], cheminformatics, including the use of ML and AI, is based on Lewis structures. Even the widespread fingerprints used for very high-throughput screening [71] are based on the occurrence of patterns within the Lewis structure.

For chemists, the Lewis structure represents both metadata for AI/ML and an essential language for communication. However, like language, the Lewis model is context dependent (aromatic bonds, boron bonding…) and relies on the interpretive knowledge of the chemist, which limits its suitability as a metadata model. Here, we return to the three languages of chemistry mentioned above. We are unaware of another branch of science in which the principal language of everyday communication in the science is “only” a model and a context-dependent one at that [72]. It is idiomatic, fuzzy, incomplete and sometimes redundant, exactly like a real written and spoken language. These properties, which embody the power of our everyday chemistry language, all hinder communication with learning machines or artificial intelligence. The consequence is that we must distinguish far more clearly between the chemical ontology of AI and ML and our everyday language.

The use of the Lewis model goes even further. Biomolecular simulations are based on molecular force fields, which are simply Lewis structures translated into a classical mechanical model that punishes deviation from preferred bond lengths, angle and dihedrals, and considers non-bonded electrostatic and van der Waals contributions. Thus, there are potentials for bonds, angles between bonds, dihedrals and interactions between distant atoms combined with a point-atomic representation [73]. These force fields have attained a remarkable level of accuracy for proteins, so that force-field based simulations have become predictive in many fields of biology, medicinal chemistry and biophysics [74].

### Models, approximations and paradigms

Despite the many advances made since chemists reached an understanding of chemical structures, there is still no all-encompassing theory to enable accurate prediction of structure–activity or structure–function relationships. Chemistry remains a confusing science in which the often overlapping concepts of models, approximations, ontologies and paradigms are blurred. Kuhn’s incommensurable paradigms [75] are easy to discern, if controversial, in physics but far less so in chemistry, where they may not even be recognizable. Bonding models are one such case.

Approximations very often become models in chemistry. The two most prominent examples are perhaps the LCAO approximation and molecular orbitals, as outlined above. Some approximations are short lived as models because their deficits quickly become apparent. Others, above all LCAO and MOs, become part of the conceptual fabric of the science because they work so well. The frequency with which necessary approximations attain the status of models is, however, perhaps unique to chemistry.

Paradigms are another matter. The majority of chemists probably cannot recognize Kuhn’s incommensurable paradigms in their subject, no more than they can identify scientific revolutions within chemistry. Halloun’s alternative view of paradigms [76] is closer to chemical reality. Halloun regards paradigms as being personal, rather than universal to the science, and allows apparently incommensurable paradigms to exist side by side in a personal paradigm. Wendel [77] characterized this change as follows:

“By atomizing and personalizing paradigms, Halloun has reduced the vision-altering, community defining character of the Kuhnian paradigm to a matter of choosing the appropriate paradigm for the situation at hand. Instead of a crisis over how scientists see the world, we have an epistemological supermarket.”

Halloun’s personal paradigm describes the community paradigm in chemistry almost perfectly. Chemists allow Lewis bonding theory, which is closely related to Valence Bond (VB) [78] theory, to coexist with MO theory, using whichever they find most appropriate for the question at hand [79]. For instance, an MO picture of the S_N_2 reaction is attractive but less common than the compact and informative “curly arrow” picture. The Diels–Alder reaction or aromaticity, on the other hand, cannot really be treated adequately within the Lewis picture without resorting to the Dewar–Zimmerman rules [80–81] or quantitative VB calculations. If we delve deeper, we find MO interpretations of, for instance, non-covalent interactions [82] coexisting with more fundamentally physical electrostatic pictures [83]. Indeed, the polarization/charge transfer shown in Figure 4 is often interpreted as donation into an antibonding σ*-orbital.

One feature with this Halloun-like situation in chemistry is that many practitioners do not recognize it for what it is, which leads to controversial discussions about which model is correct, or more precisely, more correct. This is neither constructive nor of any practical use but has led to some controversial contributions [84]. Above all, claiming that one model (usually applied incorrectly) “does not work” in order to promulgate an alternative model contributes nothing significant to chemistry but such claims are featured in the secondary literature disturbingly often [85–86]. Over and above the intellectual belief that chemistry in general needs a clarification of its approximations and models and the exact nature of chemistry paradigms, ML and AI must impose constraints and conditions on the ontology of chemistry. Conceivably, the ontology of chemistry within ML and AI could exist in parallel to the bonding models used and discussed in teaching and research. This, however, would waste a unique opportunity to rationalize chemical thinking and communication and to establish the concept of models as the language of chemistry. The major requirements of a chemical ontology for AI and ML are that it

1. can handle all the bonding and structural features necessary to achieve the required goals (i.e., that it can describe current chemistry as completely as possible),
2. is suitable for fast and efficient machine code based on existing or future line notations or other 2D representations,
3. is non-redundant in order to avoid dependencies within the ML process.

These requirements are imposed by external conditions and likely future applications. Requirement (1), for instance, must in future include supramolecular chemistry, which means that the models should be able to reproduce molecular aggregation via weak interactions. Paradoxically, exactly such interactions between drug molecules and proteins form much of the basis of classical cheminformatics. These are, however, very specific in nature and have generally been defined in detail for, for instance, scoring functions. Current models and descriptions are poorly suited for the more varied interactions involved in, for instance, self-organization in technical systems.

Requirement (2) results from the conformation problem. Any technique or model that requires a specific 3D molecular structure must deal with conformational flexibility, which introduces an additional step into the descriptor-generation process that expands exponentially with increasing size of the system. This very often rules out MO-based descriptions.

Requirement (3) pays tribute to the statistical nature of AI and ML: Dependencies between descriptors lead to poorly determined statistical modeling.

As outlined above, this will happen almost exclusively based on the Lewis bonding model. Such applications must extend the scope of the Lewis model by automatically recognizing the context-dependent features like aromaticity or a tendency to undergo electrocyclic reactions. This is nothing magical; chemists do it all the time. The most likely conclusion is that the Lewis model combined with patter-recognition techniques can do just about anything that alternative MO-based models can, even though it is a context-dependent model.

The Lewis, VSEPR, quantitative molecular orbital and two-center three-electron bond models already do quite a good job of describing the structure and chemistry of molecules, and the latter helps to describe many reactions. Extensions of these models to recognize aromaticity also exist and similar ones can be imagined for electrocyclic reactions. There is, however, a need to add non-covalent interactions to the model in order to take the importance of complexation and aggregation via non-covalent interactions into account. This could be done in a purely ad hoc fashion for each type of interaction. This is how force-field developers have generally approached the subject. Specific potential functions are added to empirical force fields for hydrogen [87] or halogen [88] bonding. However, given that researchers have been busily defining additional non-covalent interactions such as tetrel [89], pnictogen [90] and chalcogen bonding [91], this approach leads to unnecessary complication of the model. As these interactions together with hydrogen and halogen bonding can all be treated within the single “σ-hole” framework [92], a single unified approach seems possible. However, such an approach would require a wave function or electron density, which is difficult to reconcile with requirement 2 above. A possible solution would be to use computationally very efficient quantum mechanics such as, for instance, semiempirical molecular-orbital theory or tight-binding density-functional theory. As outlined above, these techniques are fast enough to be applied to entire databases [60] and can be parameterized especially to reproduce key properties such as the molecular electrostatic potential [93] or the molecular polarizability [94]. Such calculations, however, have a serious practical disadvantage in AI/ML applications to very large datasets: Quantum mechanical calculations require 3D molecular structures, which each only represent one of sometimes very many energetically accessible conformations for flexible molecules. Thus, the calculations must be preceded by extensive conformational searches and many conformations must be stored for each molecule. The seemingly less sophisticated 2D models are the answer because they consider all conformations implicitly. For instance, the 2D Gasteiger–Marsili charge model [38–39] could be extended to produce fast, approximate 2D representations of electron densities that give extrema of the molecular electrostatic potential on the van der Waals surface of the molecule, rather than the outdated and often misleading net atomic charges [95]. Simple additive models for polarizability already exist [96]. As quantitative models for intermolecular interaction energies can be built using these quantities [97], developing a ML-suitable model for intermolecular interactions is feasible. As noted above, old models such as Gasteiger–Marsili [38–39] charges or Hammett constants [59] have been used in recent applications. This development ignores the immense improvements in hard- and software of the last four decades. AI and ML mandate a rebirth of research into such techniques in a modern context.

The above discussion suggests an important role of Occam’s Razor (*lex parsimoniae*) in the design of such an ontology. Hoffmann, Minkin and Carpenter [98] have discussed the role of *lex parsimoniae* in chemistry and come to the correct conclusion that it is a philosophical but not scientific principle that should be applied with great caution, if at all, in chemistry. At the same time, they recognized the need for a model language in chemistry:

“*The facts by themselves are indigestible. They are, and must be, encased in language, connected to frameworks of understanding (theories).*”

This is what we called the everyday language of chemistry above. It is successful beyond all expectations and accounts for much of the creativity found in experimental chemistry. It is, however, pragmatic to the extent that the folk ontologies [11] or personal paradigms [76] of chemists are assembled from available models and switch happily between them. Consider, for instance, the alternative Woodward–Hoffmann [29] and Dewar–Zimmerman [80–81] rules for electrocyclic reactions. They can be used interchangeably and both give correct predictions. However, the Dewar–Zimmerman rules depend on the concepts of Hückel and Möbius aromaticity, which in turn are derived from molecular-orbital theory. In essence, the overlap arguments of Woodward and Hoffmann have been replaced by Lewis-like (plus aromaticity) principles by Dewar and Zimmerman. Thus, despite Occam, two models exist happily side by side in the everyday language of chemistry. There are many more examples.

The requirement that a chemical ontology for ML be compact and non-redundant, however, elevates the *lex parsimoniae* to a guiding principle that does not apply to everyday communication. Notably, this requirement has been advocated on purely intellectual grounds for models of chemical bonding in general [99].

But, even if we had a suitable ontology that conforms to the three requirements, is chemistry ready for big data, ML and AI? Probably not: simply because there are not yet big data in chemistry. This would require sufficient measurements under the same conditions with varying parameters, a homogeneous spread of measured properties over chemical space, and adequate standards for data measurement and reporting. None of these exists in chemistry at present. Melting points of organic compounds are an example. Melting points have been reported for millions of organic compounds, which makes them an apparently good candidate for big-data applications. However, giving melting points for solid compounds is mandatory in publications. Therefore, everybody measures melting points but they are not the primary interest. Varying purity, heating rates, and even if the experimentalist is actually looking when the compound melts, affect the results strongly. Measuring the melting point is a necessary chore. The result is that the data are of very poor quality. This can be seen in a quite recent quantitative structure–property relationship study that gave a mean square error between experiment and the models between 42 and 66K [100]. This is not useful performance and is unlikely to be the result of poor descriptors or modeling.

The situation is subjectively even worse in biology. A very relevant factor for biological data is the laboratory in which they were measured. Very good agreement between simulations and experiment can be achieved for diverse systems [101] but generally, only if the experimental data were obtained in a single laboratory, which is the case for reference [101].

The way forward probably lies in Halloun’s definition of paradigms [76]. Chemists in general, and cheminformaticians in particular, will extend the existing Lewis and VSEPR models to include necessary features to describe reactions, “non-classical” bonding etc. This is a normal process in chemistry, as shown by the introduction of three-center two-electron bonds into the Lewis picture [44] and is what Weisberg describes as a “folk ontology” [11]. This process will continue as hitherto unrecognized bonding features become apparent and the need arises to describe them in AI or ML applications. Descriptions of directional non-covalent interactions, for instance, are necessary for any applications involving crystal engineering. These can be treated adequately using anisotropic electrostatics [102] and dispersion [103]. It is, however, important to limit these extensions to models that are as parsimonious as possible but at the same time generally applicable [99]. The alternative patchwork of competing bonding models based on non-separable and non-unique interactions cannot provide a stable framework for AI and ML. In this respect, the IUPAC definition of the hydrogen bond [104] serves as an admirable example of how not to do it. It is also important to avoid competing techniques for calculating notional characteristics of interactions such as polarization and charge transfer: Because the partitioning of a single effect into these two concepts is essentially arbitrary, techniques such as NPA [22] and ALMO [23] can give results that differ by large factors for the same system. Using one of these techniques exclusively would be feasible but an unnecessary breach of the non-redundancy condition outlined above. In this respect, the requirements for a chemistry ontology for AI and ML are far more stringent than those of a folk ontology [11], which in many ways resembles Halloun’s personal paradigms [76].

Chemistry is not a solved science, so that the above process must be dynamic. Until 2007, for instance, just about all force fields and computer-aided drug design techniques treated the interaction between halogens and nucleophiles as repulsive; whereas we now know that halogen-bonding attractions can be as strong as hydrogen bonds. There will be more such examples but it is important to identify the encompassing phenomenon, rather than defining a wealth of apparently unique interactions that in reality share a common origin. This common origin is the key to successful chemistry models.

## Conclusion

Data infrastructures are evolving and growing in many areas without being interoperable, giving rise to a phase of creolization, in which many inefficiencies in working with data retard innovation [105]. Regarding chemistry as a large evolving infrastructure, with knowledge and data being generated on a daily basis, one can see not only many examples of such inefficiencies but also many practices that are still artisanal.

Chemistry needs models but chemistry also needs to recognize that models are models. The future demands of AI and ML in chemistry will require not only that data collection and storage be revolutionized but also that the predominant 2D Lewis and VSEPR bonding models be extended rationally and carefully to allow them to describe the phenomena that influence physical, chemical, biological and medicinal properties of molecules and aggregates.

Halloun’s view of paradigms [76], rather than Kuhn’s [75], appears to be most appropriate for chemistry. Existing, limping paradigms are typically extended pragmatically to encompass new situations. The personal nature of paradigms applies especially to different areas of chemistry. VSEPR, for instance, plays little or no role for most organic chemists. Indeed, these personal paradigms or folk ontologies not only vary from chemist to chemist but also over time. Chemists cherry pick their models to suit their needs. An organic chemist who uses predominantly the Lewis bonding picture or an inorganic chemist rooted in the VSEPR model switch quite happily to qualitative molecular-orbital arguments to explain electrocyclic reactions or the structure of ferrocene, even in teaching.

In contrast, cheminformatics will need to adopt a compact extended version of the Lewis/VSEPR model that relies as little as possible on arbitrary partitioning or analysis schemes and that can describe everything that needs to be described non-redundantly.

In this respect, the advent of AI and ML in chemistry can provide impetus for chemistry in general to recognize the role of models and to agree on unified, parsimonious model standards. This will not affect the everyday language of chemistry but may help understand the unique basis of chemical language.
